# Pathways to Longevity

**DOI:** 10.1093/geroni/igaf122.065

**Published:** 2025-12-31

**Authors:** Peter Martin, Bradley Willcox

## Abstract

Although life expectancy has dramatically increased over the last century, reaching the age of 100 years is still somewhat rare. Past investigations have investigated correlates of longevity, but less research has addressed different pathways to longevity. The purpose of this symposium is to highlight components distinguishing different survivorship groups. A second purpose is to assess whether longevity groups show different levels of functioning and mortality risk. All four presentations conducted latent profile analyses to determine the most optimal number of longevity groups and included additional analyses to predict functioning and mortality risks among the groups. The first presentation included longevity components based on the Georgia Centenarian Study model (e.g., family longevity, environmental support, and individual characteristics) that defined latent classes predicting levels of functioning. The second presentation used a similar set of longevity predictors in the Iowa Centenarian Study as the basis for exploring within-domain and between-domain longevity groups. The Kuakini Hawaii Centenarian Study presentation addresses the relevance of two genetic factors (i.e., APOE and FOXO3) in combination with environmental factors to distinguish between longevity groups. Finally, findings from the Health and Retirement Study display distinct profiles of longevity among adults 80 years and older based on family longevity, physical health, personality, and life satisfaction. The presentations also address profile group differences in functioning and mortality risk. The four presentations provide novel information about different profile groups of long-lived individuals, underlining policy and practical implications for these exceptional survivorship groups as they show different levels of functioning.

